# Editorial: Education in Genitourinary Surgery 2022

**DOI:** 10.3389/fsurg.2022.1107450

**Published:** 2022-12-23

**Authors:** Carolin Siech, Benedikt Höh, Felix K-H. Chun, Ulrike Necknig

**Affiliations:** ^1^ Department of Urology, University Hospital Frankfurt, Goethe University Frankfurt am Main, Frankfurt am Main, Germany; ^2^ Urologische Praxis Lindenberg, Lindenberg, Germany

**Keywords:** education, genitourinary surgery, surgery, training, curriculum - undergrad and postgrad

**Editorial on the Research Topic** Education in Genitourinary Surgery 2022

Education is a key to safe clinical practice and effective professional development. Therefore, it was our pleasure to edit and coordinate a wide range of original articles and reports within the research topic: Education in Genitourinary Surgery 2022.

In a prospective controlled study, Britz et al. reported that relying on simulation patients instead of real patients was comparable regarding the acquisition of communicative competence (e.g., taking medical histories) among undergraduate medical students.

Although the decision-making process for the choice of specialty is multifactorial, Ekrutt et al. demonstrated that a robot-assisted hands-on training course, implemented into the medical curriculum, led to an increased students' interest to turn into urology and into a surgical discipline in general.

Mentoring plays an important role in staff development. Necknig et al. evaluated the competence development by the mentee-based assessment tool for role development of interpersonal competencies in surgical professions (MatricS). In this prospective longitudinal study, mentees acquired competences in all roles and mentee self-assessments correlated well with the mentor assessments.

Limited resources and scarce operating theater capacity necessitate an increase in the effectiveness of surgical training. Assessing the time-differential between senior and junior residents in a surgical simulation of laparoscopic radical and partial nephrectomy, Eber at al. identified hilar dissection as a decisive step for the performance time regarding laparoscopic nephrectomies.

Structured curricula are demanded to improve training programs. Siech et al. demonstrated a high level of acceptance of the newly implemented residency rotation program at the University Hospital Frankfurt, including urologic practice, Intermediate Care Unit, urooncology rotation and clinical exchange.

In a retrospective analysis, Maurer et al. explored the long-term outcomes after transcorporal placement of artificial urinary sphincter systems in high-risk patients. Social continence rate was almost 80% with a mean device survival of 7 years.

As editors and coordinators of this Research Topic, we thank all the authors and reviewers for their contributions within the field. The present publications will open new perspectives in surgical education and beyond.

